# Logistical challenges and assumptions for modeling the failure of global cessation of oral poliovirus vaccine (OPV)

**DOI:** 10.1080/14760584.2019.1635463

**Published:** 2019-07-02

**Authors:** Kimberly M. Thompson, Dominika A. Kalkowska

**Affiliations:** Kid Risk, Inc., Columbus, OH, USA

**Keywords:** Polio, eradication, dynamic modeling, oral poliovirus vaccine

## Abstract

**Introduction**: The inability to successfully stop all use of oral poliovirus vaccine (OPV) as part of the polio endgame and/or the possibilities of reintroduction of live polioviruses after successful OPV cessation may imply the need to restart OPV production and use, either temporarily or permanently.

**Areas covered**: Complementing prior work that explored the risks of potential OPV restart, we discuss the logistical challenges and implications of restarting OPV in the future, and we develop appropriate assumptions for modeling the possibility of OPV restart. The complexity of phased cessation of the three OPV serotypes implies different potential combinations of OPV use long term. We explore the complexity of polio vaccine choices and key unresolved policy questions that may impact continuing and future use of OPV and/or inactivated poliovirus vaccine (IPV). We then characterize the assumptions required to quantitatively model OPV restart in prospective global-integrated economic policy models for the polio endgame.

**Expert commentary**: Depending on the timing, restarting production of OPV would imply some likely delays associated with ramp-up, re-licensing, and other logistics that would impact the availability and costs of restarting the use of OPV in national immunization programs after globally coordinated cessation of one or more OPV serotypes.

## 1. Introduction

Now nearly 20 years past the year 2000 target for global polio eradication [1], the polio endgame continues to extend beyond planned milestones and to demand more resources. Prior modeling suggested the need for globally coordinated cessation of oral poliovirus vaccine (OPV) after the eradication of wild polioviruses (WPVs) to end all cases of poliomyelitis [2], and OPV cessation became an essential component of the polio endgame in 2008 [3]. In late April-early May 2016, global cessation of all serotype 2 OPV occurred (i.e., OPV2 cessation) [4,5], but the potential need to restart the production and widespread use of OPV2 remains an issue given ongoing transmission of circulating vaccine-derived polioviruses (cVDPVs) of serotype 2 (cVDPV2s) [6]. We define OPV restart as the need to begin new bulk production of any serotype OPV, and we note that this differs from using the existing supplies of mOPV from stockpiles for outbreak response created prior to cessation of that OPV serotype. Global polio endgame modeling that assumed optimal risk management (i.e., minimal emergence risks and very high program performance) suggested a relatively low-probability (i.e., approximately 6%), but high-consequence risk of needing to restart OPV use following global OPV cessation [7]. The analysis assumed successful OPV cessation would occur due to ideal management of population immunity to transmission during the time before OPV-cessation [7], and consequently most of the risks of OPV restart (N = 52/57, 91%) in that analysis related to reintroductions of vaccine-derived polioviruses from infected individuals with primary immunodeficiencies who take longer to clear infections (i.e., iVDPVs) with the remaining 9% (N = 5/57) OPV restart events following breaches in containment [8]. A recent review discussed serotype 2 live poliovirus transmission for the first 2 years after global OPV2 cessation (i.e., April 2016-April 2018), identified the need for OPV2 restart planning, and offered insights relevant to OPV restart risks approximately 2 years after OPV2 cessation [6].

In this analysis, we go beyond discussing the risks of OPV restart [6] to identify the associated logistical challenges and support characterization of assumptions for use in prospective global-integrated economic policy models for the polio endgame. We develop a comprehensive set of scenarios and assumptions for modeling the possibility of OPV restart, and discuss the potential trade-offs of different choices. Given ongoing discussions about the future of the Global Polio Eradication Initiative (GPEI) and future GEPI strategic plans, we explore the complexities associated with completing and certifying the eradication of WPV serotypes 1 and 3, and we consider how restarting OPV might impact the future use of inactivated poliovirus vaccine (IPV).

## 2. Important serotype differences in risks

Phased OPV cessation reflects the different ends of transmission and nature of the characteristics of the three poliovirus serotypes. As summarized briefly in Table 1, differences exist between WPV transmissibility (i.e., serotypes 1 > 2 > 3) and neurovirulence (i.e., serotypes 1 > 3 > 2), OPV transmissibility (i.e., serotypes 2 > 1 > 3) and neurovirulence (i.e., serotypes 3 > 2 > 1), and the relative take rate for each OPV serotype for recipients of trivalent OPV vaccine (i.e., serotypes 2 > 1 > 3) [9,10].

**Table 1.** Serotype-specific characteristics and model inputs.

Model input	Serotype 1	Serotype 2	Serotype 3	Source(s)
Last reported WPV case	Ongoing transmission in Pakistan and Afghanistan	October 1999	November 2012	[11–13]
Average paralysis-to-infection ratio for fully susceptible individuals				[10]
- WPV	1/200	1/2000	1/1000	
- OPV	7.4×10^−8^	6.2×10^−7^	1.3×10^−6^	
Relative basic reproduction number (R_0_) for WPV or fully-reverted VDPV (relative to serotype 1)	1 (reference)	0.9	0.75	[10,14]
Relative R_0_ of OPV parent strain to WPV or VDPV	0.37	0.55	0.25	[10]
Average time (days) to revert from OPV to fully-reverted VDPV	620.5	408	620.5	[14]
Relative average per-dose OPV take rate (always 2>1>3, value within range depends on specific population) for recipient of trivalent OPV	0.583-0.875	1 (reference)	0.45-0.75	[7]

OPV: oral poliovirus vaccine; R_0_: basic reproduction number; VDPV: vaccine-derived poliovirus; WPV: wild poliovirus.

Successful OPV cessation depends critically on ensuring high population immunity to transmission prior to coordinated cessation of the OPV serotype [15]. Unfortunately, the GPEI did not fully recognize the risks of OPV evolution and the creation of cVDPVs. In the mid-2000s the GPEI started preferentially using serotype 1 or 3 monovalent OPV (mOPV1 or mOPV3) and then using bivalent OPV (bOPV, containing serotypes 1 and 3) starting in 2010 instead of trivalent OPV (tOPV, containing all three serotypes) for some supplemental immunization activities (SIAs). The preferential choice of OPV that did not contain serotype 2 for SIAs led to declines in population immunity to transmission for serotype 2, and unfortunately increased cVDPV2 outbreaks [16–18], without any benefit associated with stopping ongoing transmission of serotypes 1 or 3 [19,20], because the key challenge to eradication is the failure to vaccinate, not vaccine failure [21]. Prior to OPV2 cessation, the GPEI undertook some efforts to intensify serotype 2 population immunity to transmission by conducting tOPV SIAs in the run up to OPV2 cessation, which also offered some protection from unauthorized serotype 2 use after OPV2 cessation [22,23]. Remarkably, despite prior experience with tOPV intensification prior to OPV2 cessation and modeling that demonstrates the need to maintain high population immunity to transmission for serotypes 1 and 3 prior to their coordinated cessation [24,25], the GPEI recently used and continues to plan to use mOPV1 instead of bOPV in some high-risk areas [26]. This mOPV1 use creates immunity gaps for serotype 3 (i.e., higher risks for future opportunities for cVDPV3 outbreaks) without any expected benefits associated with accelerating eradication of serotype 1 wild poliovirus [19–21].

The risks of cVDPVs vary by serotype and the risk management activities undertaken prior to and after OPV cessation [6]. Stopping the current and preventing future cVDPV2s depends on current outbreak response efforts using mOPV2 from the OPV vaccine stockpile. Unfortunately, 2 years after OPV2 cessation, outbreak response efforts have not succeeded in stopping all serotype 2 live poliovirus transmission to date, and as the time since OPV2 cessation increases, the probability of needing to restart production and broader use of OPV2 increases. The risks of restarting OPV serotypes 1 and/or 3 after their coordinated cessation will depend on the management of population immunity to transmission in the run up to their cessation [24,25]. Therefore, delays in stopping the transmission of serotype 1 WPV combined with the absence of serotype 3 WPVs since the end of 2012, ongoing cases of VAPP caused by serotype 3 OPV, and concerns about OPV production capacity, should motivate earlier OPV3 cessation than OPV1 cessation (i.e., phased cessation of the last 2 serotypes).

Despite significant emphasis by the GPEI on the introduction of IPV into national immunization programs in all countries, IPV plays a relatively minor role in population immunity to transmission in countries characterized predominantly by fecal-oral transmission [27,28]. IPV offers susceptible individuals protection from paralysis (in the event that they become infected with a live poliovirus) and some reduction in their viral shedding, which may reduce their participation in transmission. However, receipt of IPV does not prevent individuals from contributing to transmission, and it plays little role in overall transmission of live polioviruses in the population. In addition, with poliovirus surveillance dependent on detecting individuals with paralysis, IPV use decreases the frequency of observing transmission by decreasing paralysis in the population, without the ability to stop the transmission in most populations, even with very high immunization coverage (e.g., recent experience in Israel [29]).

## 3. Trigger(s) for OPV restart

After coordinated cessation of one or more OPV serotypes, the decision to restart OPV will not come lightly, and modeling OPV restart will benefit from discussions related to establishing specific criteria and a trigger for deciding whether and when to restart OPV. The actual decision to restart OPV will likely require a resolution at the World Health Assembly level that the transmission of cVDPV2s (or a reintroduced WPV2 or other OPV2-related live poliovirus) is expected to (or has) become established and cannot be stopped except with the reintroduction of OPV2 into routine immunization (RI) and for use in outbreak response SIAs.

Prior prospective global integrated economic policy modeling of the polio endgame used a threshold of 50,000 total paralytic polio cases for all three serotypes as a criteria for restarting trivalent OPV for the base case analysis, while demonstrating the increased number of OPV restarts implied by thresholds as low as 1,000 total cases in the context of sensitivity analyses [7]. That modeling assumed optimal risk management by countries and the GPEI (i.e., minimal emergence risks and very high program performance), and used a very simplistic approach to model OPV restart (i.e., restart of tOPV use and abandoning OPV cessation as a global strategy to return to control upon reaching the threshold) [7]. However, given the current conditions, more realistic assumptions are needed to better model OPV restart and the different nature of the risks of the three serotypes. Phased cessation of the OPV serotypes suggests the possibility of using serotype-specific thresholds for modeling (e.g., 1,000 or 5,000 cases to restart a serotype-specific OPV, which could vary by serotype). After OPV cessation, any paralytic cases caused by a live poliovirus (i.e., even a less than fully reverted OPV) would count toward the total. While the actual decisions to restart OPV production and use in RI will not likely involve a numerical trigger, given the numerous pathways in a stochastic model that could lead to uncontrolled transmission, using a consistent numerical threshold (or serotype-specific thresholds) provides a modeling strategy that automates prospective action [7]. Sensitivity analyses around the selected threshold(s) can then reveal the different paths that lead to restart and the different types of errors (i.e., restarting bulk OPV production when the transmission could actually have been stopped with available supplies or allowing large burdens of cases to accumulate prior to restarting).

The ability to quickly restart production and use of an OPV serotype will likely depend on the existence of any ongoing production of at least one licensed OPV vaccine. Currently, the number of OPV vaccine manufacturers continues to decline as OPV demand decreases (i.e., in addition to manufacturers in a small number of self-producing countries), and only three manufacturers continue to produce OPV for the global market as of early 2019. One of these manufacturers will likely stop its bulk production of all OPV by the end of 2019, although it will continue to fill its bulk OPV until at least until 2021, and another one only recently started to supply the global OPV market. With complete OPV cessation, OPV vaccine manufacturing and support of licensed OPV products will end.

Up through 2021, restart of OPV2 in the form of tOPV could occur, albeit with some delay. Specifically, the primary OPV manufacturer that could still produce OPV2 and continues to produce bOPV, could use existing bulk of OPV2 and fill tOPV. However, the time delay to obtain these vaccines and the availability of vaccines in self-producing countries will require management. Notably, in the context of complying with containment requirements for serotype 2 live polioviruses [30], self-producing countries have already destroyed or will soon destroy any supplies of OPV2 that they produced prior to OPV2 cessation. In addition, while the initial production may not support full catch-up of cohorts that missed OPV2 since mid-2016, depending on the timing of the decision to restart tOPV, these cohorts could most likely be covered within a couple of years.

Longer term (i.e., after 2021), if one or more existing manufacturers maintains OPV production capacity, then adding the restarted OPV strain could occur relatively quickly (e.g., 3 years). Restarting OPV would incur time delays associated with the production of new bulk vaccine for the restarted serotype and for filling, finishing, re-licensing, and distribution of the restarted OPV product. In contrast, if OPV vaccine production stops (i.e., no serotypes of OPV are in production); then, the delay to restart bulk OPV production will require additional years to build new capacity (e.g., 7 years for the first OPV serotype, and 5 years for each additional OPV serotype). In the event of an OPV restart, in theory any Sabin IPV manufacturer(s) could potentially use the OPV that they produce (prior to inactivation) to license, fill, and finish an OPV product that could accelerate the OPV restart, particularly since the production of Sabin IPV requires the equivalent of multiple doses of OPV per one IPV dose. However, this will require some time (for the licensing, filling, and finishing), and it would effectively remove IPV production capacity from the supply chain. Currently, we assume that no Sabin IPV manufacturers will license their OPV for use unless an OPV restart occurs, which will increase market demand for OPV and decrease demand for IPV.

In the event of an OPV restart, depending on the timing and serotype, the restarted OPV could potentially use a new OPV (nOPV) strain, that may better resist reversion [31], although the development of serotype 2 nOPV may become a real option once available [32], perhaps as early as 2021, with nOPVs for serotypes 1 and 3 lagging further in time. The decision to restart OPV would also likely require and come with the end of the GAPIII containment requirements [30] for the restarted OPV, since at that point transmission of the live polioviruses of the restarted serotype would indicate no need for containment. With the lifting of containment requirements, additional OPV vaccine manufacturers could enter the market, although their OPV products would most likely become available after 5–7 years of delay.

The nature of the OPV restart will depend on perceptions about the overall strategy of OPV cessation as a requirement for the polio endgame and the global commitment to ending all cases of poliomyelitis. If an OPV restart occurs in the context of a global effort to boost population immunity to transmission for serotype 2 sufficiently such that a second globally coordinated cessation of OPV2 can occur; then, this would imply OPV restart as a temporary measure that would impact the attractiveness of (re)entry into the OPV market for manufacturers.

## 4. Serotype(s) of OPV to restart under different conditions

Consistent with the current national polio vaccine strategies, we assume that countries will likely make different choices about whether and how to use OPV in their national immunization programs in the event of an OPV restart. With respect to globally modeling national immunization policies for the polio endgame, we assume that at the time of all OPV cessation, any high- and upper-middle-income countries still using any OPV will stop OPV use and switch to a national immunization schedule that includes three doses of IPV only, most likely increasingly delivered in a combination vaccine product. In contrast, we assume that until the time of coordinated cessation of the final OPV, any OPV-using countries would continue to use at least two doses of OPV containing all of the serotypes not yet stopped and at least one dose of IPV in their national immunization schedules. Due to the WHO SAGE recommendation that all countries include ‘at least two doses of IPV in their routine immunization schedule’ for at least 10 years after OPV withdrawal [33], we assume that all countries that use just one dose of IPV at the time of globally coordinated cessation of the last OPV serotype will increase then to two doses of IPV in their national immunization programs.

The polio vaccine strategy in effect at the time of restarting one or more serotypes of OPV will impact the choice of the restarted OPV vaccine. For example, if successful OPV cessation occurred for all three serotypes and cases of one serotype triggers an OPV restart; then, the restart may involve only the mOPV of that serotype. However, triggering of more than one serotype may lead to the restart of the associated OPV containing the two restarted serotypes or restart of tOPV. If the decision to restart OPV2 occurs while use of the current bOPV (i.e., serotypes 1 and 3) continues; then, OPV restart would imply restarting the use of tOPV or use of bOPV+mOPV2. Some possibility exists that this decision would come with a global statement of a failure of the OPV-cessation strategy, and lead to global production of tOPV that would allow countries to choose to use tOPV for their national immunization programs instead of some or all doses of the relatively much more expensive IPV vaccine. In the event that phased OPV cessation occurs for serotype 3 (i.e., a bOPV-mOPV1 switch), the decision to restart serotype 2 or 3 OPV could lead to a bOPV that contains both serotype 1 and the other restarted OPV, to restart of tOPV, or to use of two separate mOPVs.

**Figure 1.**
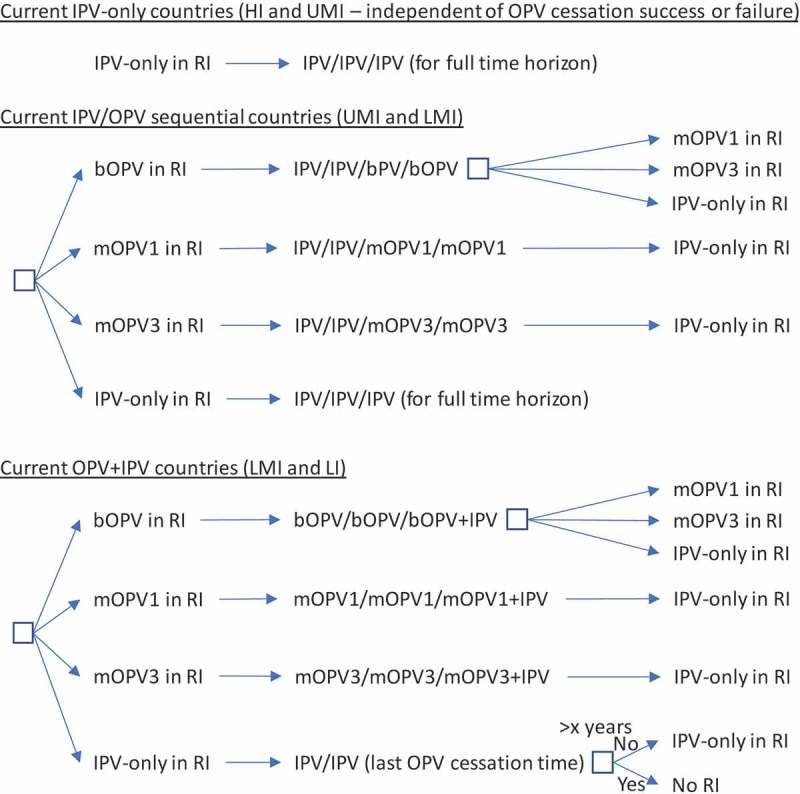
Routine immunization (RI) options for successful-phased OPV cessation.

The decision tree in Figure 1 shows the options for RI by current immunization strategy (for high-income (HI), upper-middle-income (UMI), lower-middle-income (LMI), and low-income (LI) countries) assuming a successful-phased OPV-cessation strategy (i.e., no OPV restart) and beginning with bOPV in RI for OPV-using countries, which represents the *status quo* as of early 2019. As shown at the top of Figure 1, we assume that all countries currently using IPV-only for RI will continue to do so for the 40-year time horizon of 2019–2058. The middle of Figure 1 shows the RI schedule for countries currently using an IPV/OPV sequential schedule, and the options for phased cessation of the remaining OPV serotype(s). Reaching an option on the far right that corresponds to an option that appears as an option in the initial decision (i.e., the left-most box) implies the need to follow that initial option and any subsequent ones until no further changes in the options occur (i.e., until reaching IPV/IPV/IPV (for full-time horizon)). The bottom part of Figure 1 shows the RI schedule for countries currently using an OPV schedule with one dose of IPV (i.e., OPV+IPV) and the options for phased cessation of the remaining OPV serotype(s). As above, reaching an option on the far right that corresponds to an option coming off of the initial decision (i.e., the left-most box) implies the need to go back and follow that option and any other options until no further changes in the options occur (i.e., until reaching IPV/IPV (for x years since cessation of the last OPV)) followed by No RI. As implied by the bottom of Figure 1, we assume that these countries will use a two-dose IPV schedule at the time of cessation of the last OPV serotype.

**Figure 2.**
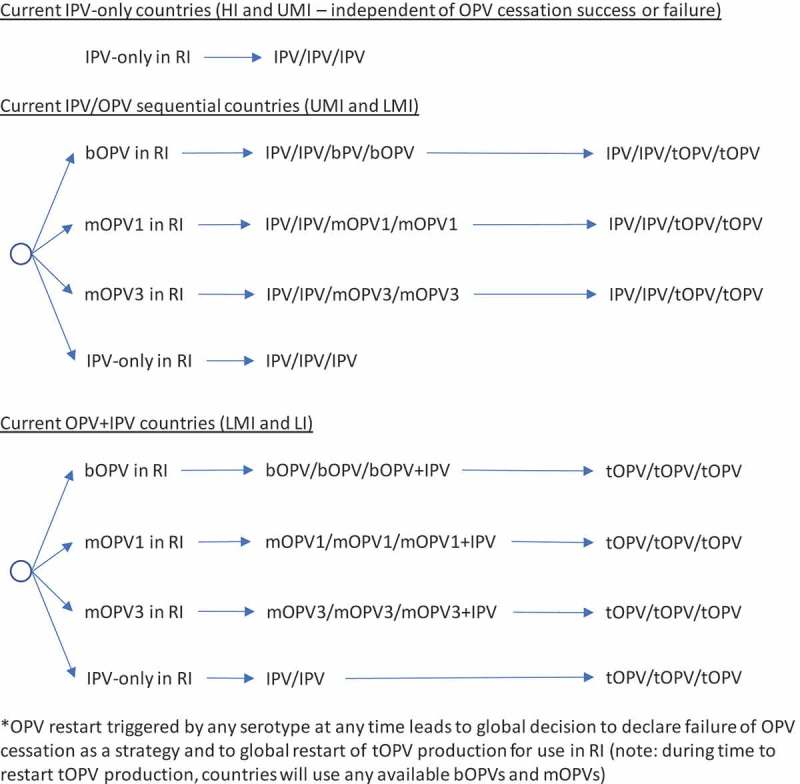
Routine immunization (RI) options with failure of the OPV-cessation strategy (control).

In the context of OPV2 restart, the options for RI become complicated quickly. As highlighted earlier, a significant issue that will arise with OPV restart will center on whether the global intention of restart comes with a statement of failure of OPV cessation as a polio endgame strategy or with an expectation that the world will again seek to stop all transmission of live polioviruses and again stop the production and use of the restarted OPV. The decision tree in Figure 2 shows the options for RI by current immunization strategy assuming that an OPV restart occurs and that this decision will end all consideration of the strategy of OPV cessation, eliminate containment requirements for live polioviruses, lead to the restart of tOPV, and suggest implicit acceptance of any future VAPP or VPDV cases associated with the use of OPV in national immunization programs (i.e., failure of the OPV-cessation strategy and return to control [7]). As in Figure 1, RI for current IPV-using countries remains unchanged. As shown in Figure 2, the decision to restart any OPV serotype after its global cessation leads to the restart of tOPV production, which OPV-using countries either use in a sequential schedule (middle of Figure 2) or revert to a three-dose tOPV-only schedule (bottom of Figure 2). Prior modeling, which triggered OPV restart after reaching a threshold of 50,000 total global cases (summed over all serotypes) [7], assumed an approach that corresponded to Figure 2.

**Figure 3.**
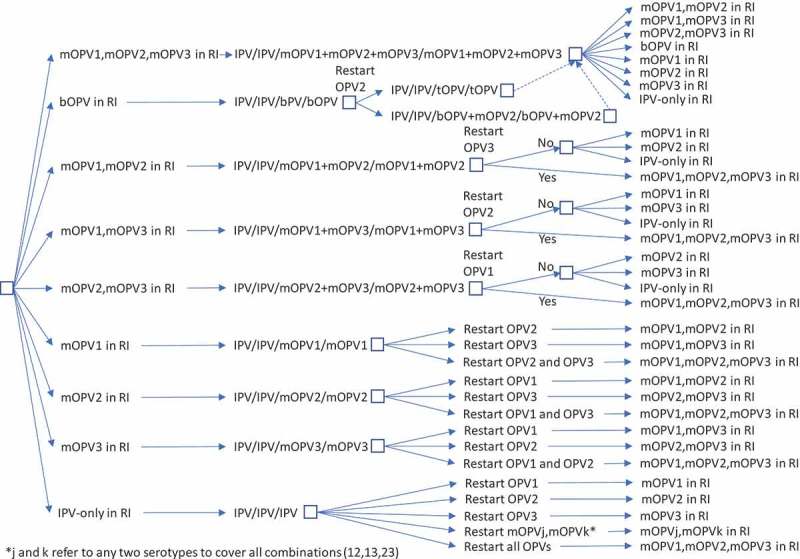
Routine immunization (RI) options for IPV/OPV countries with OPV restart and continued phased OPV cessation (starts at bOPV in RI as of mid-2019).

**Figure 4.**
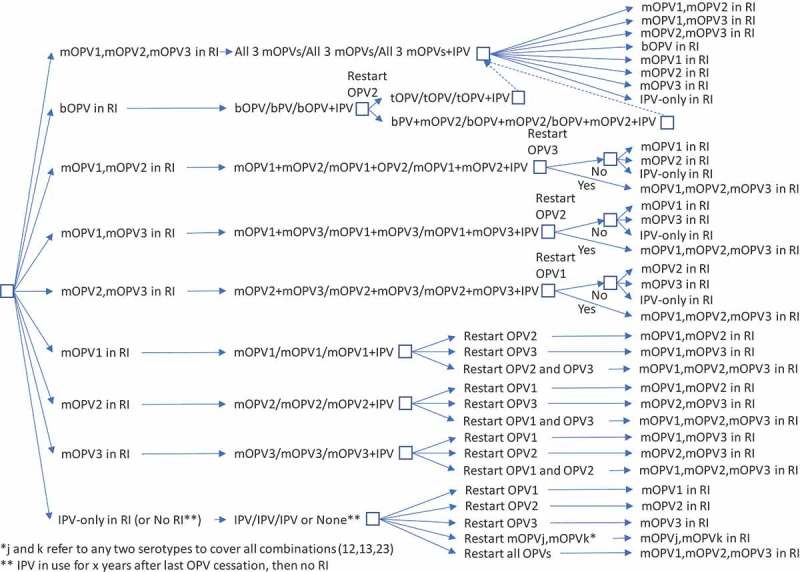
Routine immunization (RI) options for OPV+IPV countries with OPV restart and continued phased OPV cessation (starts at bOPV in RI as of mid-2019).

While Figures 1 and 2 represent the extreme policies, much more nuanced strategies also exist. For example, the decision to restart one or more previously stopped serotypes of OPV could lead to a temporary setback that does not change the polio endgame strategy of OPV cessation. Figures 3 and 4 show the options for RI for a continued policy of pursing OPV cessation in the context of OPV restart. For Figures 3 and 4, we only include current vaccines (i.e., those produced now or in recent years: tOPV, bOPV, mOPV1, mOPV2, and mOPV3), because we assume that manufacturers will not incur the costs of licensing new combinations of OPVs (although these could be added and would increase the branches of the tree for any options with more than one serotype). We included the options of all three mOPVs at the tops of Figures 3 and 4 because this option theoretically exists, and the current *status quo* of bOPV in RI appears under it and leads to the same set of decisions on the far right, as indicated by a box with a dashed line pointing to the box above it. For Figures 3 and 4, as before, many options on the right correspond to the need to go back to the left and follow a new branch, and the options on the right imply different decisions related to phasing of OPV cessation and different decisions to restart OPV. All of the decisions in Figures 3 and 4 include OPV restart, but if no OPV restart occurs, then RI will revert to the appropriate line in Figure 1.

Figure 3 shows the complexity of all of the possible types of OPV restart that can occur and all of the potential RI schedules and pathways for countries currently using an IPV/OPV sequential schedule and ultimately leading to an IPV/IPV/IPV schedule (if successful OPV cessation occurs despite one or more OPV restart events during the time horizon). Figure 3 implies that all countries would restart a sequential schedule in the event of OPV restart, although we note that some of the countries already using IPV-only RI schedules might not opt to return to a sequential schedule. To date, Israel is the only country to stop all OPV use in RI (i.e., switch to an IPV-only RI schedule) and then reintroduce OPV vaccine back into RI following restarted transmission due to a WPV importation [34]. Figure 4 shows the same for countries that currently use OPV schedules with an IPV dose given at the same time as the third OPV dose (i.e., OPV+IPV schedule). The countries following Figure 4 ultimately go to an IPV/IPV schedule for x years followed by no RI (if successful OPV cessation occurs despite one or more OPV restart events during the time horizon, with the change to no RI if x years after the last serotype of OPV cessation occurs before the end of the time horizon). Figure 4 assumes that these countries will only use IPV for the minimum years required after cessation of the last OPV serotype, for which we assume x = 10 years corresponds to current GPEI recommendations [33]. At the bottom of Figure 4, the time of IPV use for the minimum recommendation starts at the time of the last OPV cessation, and as long as the time is less than x (i.e., <x), IPV/IPV use continues.

Collectively, Figures 1–4 hint at the numerous possible decisions and pathways that could occur and the challenges of modeling OPV restart for the polio endgame. With most high- and upper-middle-income countries already using IPV-only for their national immunization programs, we assume that these countries would not restart using OPV, except for using the appropriate mOPV for outbreak response (if needed and available), or a bOPV or tOPV containing the needed serotype if the required OPV is only available in that form. In the event of OPV restart, we assume that all countries would use the appropriate mOPV in the event of an outbreak, although in the context of a rare outbreak in a relatively high-income countries, the country will likely try to stop the outbreak first using IPV since that will be the vaccine already available in the country. We also assume that any outbreak response would target all new birth cohorts born since the serotype-specific OPV cessation and include at least two high-quality rounds (i.e., not including small or poor-quality rounds that may also occur as part of the outbreak response). As implied in Figure 2, if OPV restart leads to a global declaration of failure of the strategy of OPV cessation and a shift back to tOPV use; then, we assume that low- and lower-middle-income countries will stop using IPV and use tOPV as soon as it becomes available. We assume that after OPV cessation, countries would not include preventive SIAs using OPV in their national immunization programs. However, we assume that OPV restart would include catch-up SIAs for the missed birth cohorts.

## 5. Factors affecting future OPV use

Several factors will impact the vaccine that countries use in the polio endgame. First, following OPV cessation and up to the time of a decision to restart a stopped serotype of OPV, the only OPV of that serotype available for outbreak response will reside in whatever stockpiles exist. The creation of these OPV stockpiles will occur prior to cessation, with fixed amounts of bulk and filled vaccine available with varying shelf-lives. Management of the stockpile will play a critical role in the polio endgame [6,35,36]. If demands from the stockpile exceed the available filled doses; then, shortages may lead to delays in outbreak response, which in turn may lead to a vicious cycle of increased cases, further increased demand, and more delays [6,35,36]. However, since filled vaccine expires, unused filled doses imply wasted resources.

If a decision occurs to restart one or more serotypes of OPV, the world will likely need to find a way to prioritize, ration, and/or extend the distribution of any OPV doses remaining in the stockpiles (e.g., delivering one instead of two drops per OPV dose used in outbreak response) and continue these activities for those new doses produced prior to production capacity reaching the point of full coverage. This could lead to discussion about saving doses in the stockpile for future use in RI instead of using them for outbreak response now. However, the failure to use doses from the stockpile to stop ongoing transmission creates conditions likely to lead to OPV restart. Depending on availability, we assume that the first use of the limited OPV doses in the stockpiles will go for reactive outbreak response SIAs, and not preventive SIAs or RI in areas at risk. We also assume that restarting production of OPV will lead to restarting its use in RI because the additional production will imply expected large-scale use that cannot be contained.

In the event that an OPV restart occurs with the concept of using the restarted OPV to re-eradicate the circulating live poliovirus and then again stopping the use of that OPV, as discussed above we anticipate that OPV manufacturers would likely choose to re-license the mOPV and not to add the restarted OPV to any existing OPV products (i.e., not to restart production of tOPV in the event of an OPV2 restart while bOPV use continues and not to make a new bOPV (e.g., for serotypes 1 and 2 if OPV2 restart occurs after cessation of OPV3)). Use of mOPV from the stockpiles or use of multiple OPV products in national immunization schedules after an OPV restart would imply additional costs for administration (for the multiple doses) and will raise questions about delivery of the doses at the same time or on separate encounters. OPV doses delivered at the same time may lead to lower efficacy in individual vaccine recipients due to potential interference between the OPV serotypes, with the offset of greater chance for increasing population immunity due to secondary transmission of more serotypes in the population, higher coverage, and reduced burden on the population and vaccine delivery system due to fewer encounters. Countries will likely consider using national funds budgeted for IPV to instead deliver OPV given the lower costs of vaccine procurement and administration for OPV.

## 6. Implications for prospective global-integrated economic policy models for the polio endgame

Prospective global-integrated economic policy models may stratify countries as components, or divide the world using other assumptions (e.g., approximately equal size epidemiological blocks [7]). Table 2 summarizes assumptions for use in prospective global-integrated economic policy modeling stratified using blocks, although they should also support modeling each country separately then aggregating to the global level, to characterize the impacts of OPV restart. As the polio endgame evolves, the assumptions should change as well, but in the absence of established policies, Table 2 provides a starting point. For example, if Sabin IPV manufacturers license the OPV that they manufacture for use as OPV in one or more different formulations (i.e., one or more mOPVs, bOPVs, or tOPV); then, this could significantly decrease the time for introduction of an OPV product that they might produce and offer in the future. However, we currently assume that Sabin IPV manufacturers will not incur the upfront costs associated with licensing OPV products, because currently, they would need to pass on these costs to consumers by increasing the price of their IPV, and the market is already highly sensitive to IPV price. Similarly, as investments in research and development lead to potential new vaccine options (e.g., nOPV strains), the assumptions in Table 2 may need to change with respect to the choice and properties of the vaccines available for use. Table 2 includes a toggle for this as a reminder of the need to consider future vaccines.

**Table 2.** Summary of OPV-related assumptions for prospective global-integrated economic policy models.

Model input	Value
Serotype-specific threshold number of cases since serotype-specific-OPV cessation to trigger restart of OPV for that serotype	5,000
Number of years required to fully restart OPV production while production of any licensed OPV still occurs and manufacturers maintain a stockpile of the appropriate mOPV for oSIAs	2
Number of years required to fully restart OPV production while manufacturers maintain a stockpile of the appropriate mOPV for oSIAs but have stopped production of OPV vaccine	3
Number of years required to fully restart OPV production of the first serotype (or to simultaneously restart production of more than one OPV serotype (e.g., bOPV or tOPV)) when no OPV remains available	7
Number of years required to fully restart OPV production of an additional serotype after OPV restart of another serotype already occurred	5
Minimum number of years of IPV use after cessation of the last OPV serotype (x)	10
Toggle for the availability of nOPV in the event of a restart	False
Toggle for resuming oSIAs in the event of a restart	True
Toggle for resuming pSIAs in the event of a restart	False
Toggle for continuing the global strategy of OPV cessation	Policy choice (True or False)
Type of restarted vaccine: post OPV2 cessation after detecting circulating serotype 2	tOPV or mOPV2 (+bOPV)
post OPV2 and post OPV3 cessation after detecting circulating serotype(s):	
○ serotype 2	tOPV or mOPV2 (+mOPV1)
○ serotype 3	tOPV or mOPV3 (+mOPV2)
○ serotype 2 and serotype 3	tOPV
post all OPV cessation after detecting circulating serotype(s):	
○ serotype 1	tOPV or mOPV1
○ serotype 2	tOPV or mOPV2
○ serotype 3	tOPV or mOPV3
○ serotype 1 and serotype 2	tOPV or mOPV1+mOPV2
○ serotype 1 and serotype 3	tOPV or bOPV or mOPV1+mOPV3
○ serotype 2 and serotype 3	tOPV or mOPV2+mOPV3
○ serotype 1 and serotype 2 and serotype 3	tOPV

bOPV: bivalent OPV; IPV: inactivated poliovirus vaccine; mOPV: monovalent OPV (mOPVx where x = 1, 2, or 3, corresponds to serotypes 1, 2, or 3, respectively); oSIAs: outbreak response SIAs; nOPV: new oral poliovirus vaccine; OPV: oral poliovirus vaccine; pSIAs: planned SIAs; R_0_: basic reproduction number; SIAs: supplemental immunization activities; tOPV: trivalent OPV; VDPV: vaccine-derived poliovirus; WPV: wild poliovirus.

**Figure 5.**
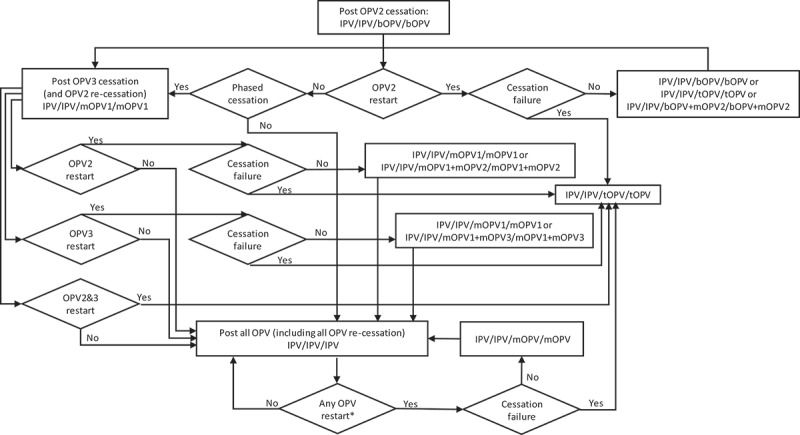
Routine immunization (RI) flowchart for blocks in the global model currently using IPV/OPV sequential schedules.

**Figure 6.**
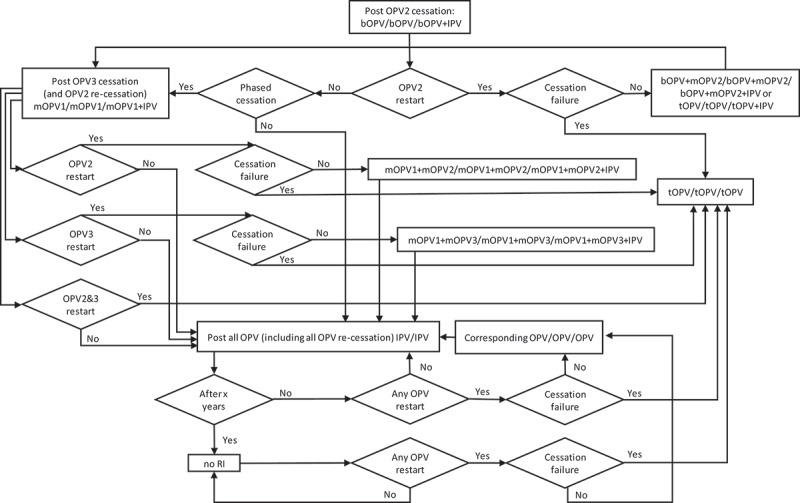
Routine immunization (RI) flowchart for blocks in the global model currently use OPV+IPV schedules.

For purposes of modeling, we also need to make assumptions about how exactly to handle OPV restart. While Figures 1–4 provide insights about the complexity of the options, we assume that modeling will require a simplified and hybrid approach. Figures 5 and 6 provide flowcharts that summarize the assumptions that we believe represent reasonable starting points for the decisions and logic required to model RI schedules for the remainder of the polio endgame starting from 2019, and allowing for any serotype of OPV restart and a decision at the time of any OPV restart about continuing OPV cessation as a polio endgame strategy (or not). We make several simplifications in Figures 5 and 6, including the most likely order for phased cessation of OPV3 next given ongoing transmission of WPV1 as of early 2019, re-cessation of restarted OPVs at the same time as one or more of the never previously stopped types, and recognition that IPV/OPV sequential schedule countries probably will not restart use of a previously stopped OPV (i.e., they will rely only on protection from IPV) unless OPV restart is required to stop transmission (see note at the bottom of Figure 5). The flowcharts in Figures 5 and 6 start at the top (with the *status quo* RI), and remains in the current place until an event occurs. Events lead to movement through the flowchart according to the arrows and decisions/outcomes (‘Yes’ or ‘No’). As shown in the boxes, in some cases, the RI vaccine option used will depend on vaccine availability, with the potential options separated by ‘or.’ With any OPV restart, the flowcharts include the global decision to continue OPV cessation as a strategy (or not), which we assume will represent a global decision driven by OPV+IPV countries (and not affecting any IPV-only countries or all (if any) IPV/OPV countries). Figures 5 and 6 allow for OPV restart for one or more serotypes (e.g., restart of OPV2 and OPV3) at the same time.

## 7. Expert opinion

The potential need to restart OPV2 production and use increases as the GPEI continues to detect the transmission of serotype 2 live polioviruses with increasing time since globally coordinated OPV2 cessation. Immediately following OPV2 cessation, the GPEI and some countries affected by cVDPV2s demonstrated hesitancy about using mOPV2 to stop outbreaks. While some of the outbreak responses succeeded (e.g., Syria) by achieving sufficiently high coverage despite significant challenges, others occurred with substantial delay and significant issues in quality (e.g., Democratic Republic of the Congo). As we noted in 2017 [36], delays in mOPV2 responses increase the risks of not controlling the outbreak [20] and creating the future need for more use of mOPV2 at a later time (as global population immunity to transmission for serotype 2 declines further) and the risks of mOPV2 itself potentially sustaining transmission in the population increases. The failure to use OPV while we can most safely do so increases the risks of needing to restart its broader use later. As we noted in 2018 [6], efforts by the GPEI to scale back on its investments in programmatic activities in key countries is already leading to diminished capacities and performance of both preventive and reactive activities that still need to occur for a successful polio endgame. As of the beginning of 2019, we still are not done with polio eradication.

Reflecting on prior perspectives, we offer our current five-year view. In 2012, we recognized the need to resolve uncertainties about potential low-cost IPV [37]; however, IPV costs remain relatively high, and uncertainty remains about the potential for significant cost decreases as a function of time. In 2014, we expected the certification of the world as free of serotype 3 wild poliovirus by 2018, and we hoped WPVs and cVPDV2 would be successfully eradicated [38]. Unfortunately, delays and poor implementation of strategies continue to increase the costs of polio eradication and complicate the polio endgame. In 2017, we expected that within the next few years, global population immunity to serotype 2 transmission would continue to decrease and the size of cohorts with no serotype 2 vaccine protection would accumulate [36]. As of early 2019, the OPV2 cessation that occurred in early 2016 led to the end of transmission of serotype 2 live polioviruses in most places, and thus declining population immunity to transmission for serotype 2. However, cVDPV2 outbreaks and the use of mOPV2 to stop these outbreaks since mid-2016 led to new transmission and variable population immunity in the outbreak areas (and potentially spilling over to some other areas). For OPV2 cessation to succeed, the GPEI must rapidly stop all serotype 2 live poliovirus transmission everywhere and contemporaneously. Unfortunately, at this time, we do not see a comprehensive GPEI plan in place to accomplish this (i.e., defined processes and funding mechanisms exist for responding to outbreaks after they occur, and support for some national programs continues (e.g., in Nigeria), but as of the time of writing, no plan exists that will get in front of and permanently stop and prevent serotype 2 transmission). We hope that such a plan will emerge and that it will succeed, but in the absence of effective GPEI management of serotype 2, we suspect that a decision to restart OPV2 may occur. Either way, in the next five years, we will know whether we will need to restart the production and use of OPV2 to stop or control transmission of serotype 2 live polioviruses. We also expect that in the next five years, the world will likely stop OPV3 use, unless world leaders declare the failure of OPV2 cessation and failure of OPV cessation as a strategy, and they decide to revert to control with tOPV.

The 2017 WHO SAGE recommendation to countries of including ‘at least two doses of IPV in their routine immunization schedule’ for at least 10 years after OPV withdrawal [33] may lead to significant costs for the polio endgame, and we anticipated in 2018 that national governments would continue to evaluate their commitments to IPV vaccination [6]. We still continue to expect that national governments will evaluate their willingness-to-pay and desire to use IPV vaccination. During the next five years, some additional manufacturing capacity of IPV may lead to sufficient supplies to support two-dose routine immunization schedules, but we expect that coverage in routine immunization will remain low in many countries, and that IPV use will remain highly uneven and IPV costs will not decline significantly. We also expect that decisions by Gavi to support (or not) the costs of IPV use in Gavi-eligible countries during the next five years will impact the decisions by countries and the global market for polio vaccines.

In 2018 we also anticipated that over the next five years the GPEI may dissolve and the GPEI partners might establish a new structure and entities to manage polio endgame risks (or not) [6]. Given the continued need for coordination due to the lack of success in polio eradication, we now expect that the GPEI will not dissolve until at least 2023, consistent with the GPEI recent new strategic plan for 2019–2023 [39].

Finally, we anticipate resolution within the next five years of the success or failure in development of viable nOPV candidates, and that GPEI partners will evaluate these candidates for potential use in outbreak response (e.g., as part of a stockpile) and/or use as OPV in routine immunization in the event of OPV restart (presumably instead of IPV). Some manufacturers of nOPV might also potentially evaluate these as IPV seed strains that would reduce containment risks associated with Sabin IPV production.

## Article highlights

- Delays in polio eradication continue to increase the chances of needing to restart OPV2
- Restarting OPV is complicated by phased OPV cessation of different serotypes
- OPV producers continue to leave the market in response to decreasing demand for OPV
- Decisions about OPV restart will likely impact the IPV market
- The development of new OPV strains may change future polio vaccine policies
